# Functionalized KIT-6/Polysulfone Mixed Matrix Membranes for Enhanced CO_2_/CH_4_ Gas Separation

**DOI:** 10.3390/polym12102312

**Published:** 2020-10-09

**Authors:** Thiam Leng Chew, Sie Hao Ding, Pei Ching Oh, Abdul Latif Ahmad, Chii-Dong Ho

**Affiliations:** 1 Department of Chemical Engineering, Universiti Teknologi PETRONAS, Seri Iskandar 32610, Perak, Malaysia; ding.sie_g03642@utp.edu.my (S.H.D.); peiching.oh@utp.edu.my (P.C.O.); 2 CO_2_ Research Centre (COSRES), Institute of Contaminant Management, Universiti Teknologi PETRONAS, Seri Iskandar 32610, Perak, Malaysia; 3 School of Chemical Engineering, Engineering Campus, Universiti Sains Malaysia, Nibong Tebal 14300, Pulau Pinang, Malaysia; chlatif@usm.my; 4 Department of Chemical and Materials Engineering, Tamkang University, New Taipei City 25137, Taiwan; cdho@mail.tku.edu.tw

**Keywords:** KIT-6, functionalization, mixed matrix membranes, CO_2_, CH_4_

## Abstract

The development of mixed matrix membranes (MMMs) for effective gas separation has been gaining popularity in recent years. The current study aimed at the fabrication of MMMs incorporated with various loadings (0–4 wt%) of functionalized KIT-6 (NH_2_KIT-6) [KIT: Korea Advanced Institute of Science and Technology] for enhanced gas permeation and separation performance. NH_2_KIT-6 was characterized by field emission scanning electron microscope (FESEM), X-ray diffraction (XRD), Fourier transform infrared (FTIR), and N_2_ adsorption–desorption analysis. The fabricated membranes were subjected to FESEM and FTIR analyses. The effect of NH_2_KIT-6 loading on the CO_2_ permeability and ideal CO_2_/CH_4_ selectivity of the fabricated membranes were investigated in gas permeation and separation studies. The successfulness of (3-Aminopropyl) triethoxysilane (APTES) functionalization on KIT-6 was confirmed by FTIR analysis. As observed from FESEM images, MMMs with no voids in the matrix were successfully fabricated at a low NH_2_KIT-6 loading of 0 to 2 wt%. The CO_2_ permeability and ideal CO_2_/CH_4_ selectivity increased when NH_2_KIT-6 loading was increased from 0 to 2 wt%. However, a further increase in NH_2_KIT-6 loading beyond 2 wt% led to a drop in ideal CO_2_/CH_4_ selectivity. In the current study, a significant increase of about 47% in ideal CO_2_/CH_4_ selectivity was achieved by incorporating optimum 2 wt% NH_2_KIT-6 into the MMMs.

## 1. Introduction

Polymer membranes have emerged as a potential candidate in carbon dioxide removal due to their economical alternatives to conventional separation processes. Membrane technology exhibits operational flexibility, small design, cost efficiency, easy scale-up, high product quality, and a small footprint [1,2,3]. However, polymer membranes commonly suffer from a trade-off between permeability and selectivity [4,5,6,7,8,9,10].

In view of the drawbacks of polymer membranes, which limit the application of polymer membranes in gas separation, development of mixed matrix membranes (MMMs) for gas separation applications has been gaining popularity among researchers in recent years. MMMs are commonly formed by incorporating inorganic fillers into the polymer matrix. Among the various inorganic fillers, mesoporous silicas appear to be a highly potential filler in the MMMs for efficient gas permeation and separation [11]. Mesoporous silicas possess several advantages including high specific surface area, high mechanical and thermal stability, and high CO_2_ adsorption capacity [12]. In addition, the high porosity of mesoporous silica facilitates gas permeation during gas separation [13].

KIT-6 (KIT: Korea Advanced Institute of Science and Technology) is a type of mesoporous silica, which is a highly potential filler for MMMs due to its large pore size. KIT-6 has drawn the attention of researchers for gas permeation and separation. Owning to the large pore size, KIT-6 is granted with an interpenetrating pore system, which enables the formation of an intimate structure with polymer matrix [4]. Intimate structure in MMMs is essential for high gas permeation and separation performance of MMMs.

The functionalization of mesoporous silica by amine groups is one of the methods to further enhance the gas permeation and separation performance of MMMs [14]. Wu et al. [14] found that by using polyethylenimine-functionalized-MCM-41 [MCM: Mobil Composition of Matter], better interfacial interaction could be formed with the polymer compared to unfunctionalized MCM-41. The amine-functionalized-MCM-41 resulted in improvement in CO_2_ permeability and CO_2_/CH_4_ selectivity as compared to the MMMs incorporated with unfunctionalized MCM-41 [14]. Meanwhile, Kim and Marand [11] performed amine functionalization on MCM-41 filler, which resulted in great compatibility with glassy polysulfone (PSF) membrane and significantly enhanced CO_2_/CH_4_ selectivity of the MMMs [11]. Furthermore, Waheed et al. [1] reported that good dispersion and adhesion of the filler with the PSF polymer were achieved by functionalizing the rice husk silica (RHS) filler with 4-aminophenazone. The MMMs incorporated with amine-functionalized RHS displayed significantly higher CO_2_/CH_4_ selectivity as well as CO_2_/N_2_ selectivity [1]. On the other hand, Khan et al. [13] reported similar results where MCM-41 was functionalized with aminopropyltrimethoxysilane. The use of amine-functionalized-MCM-41 as a filler resulted in the elimination of voids as well as good dispersion of the filler in the MMMs along with significantly higher CO_2_/CH_4_ and CO_2_/N_2_ selectivities [13]. Thus, amine functionalization on the mesoporous silica filler would significantly improve the structure of the MMMs by reducing or eliminating the void formations in MMMs, which will then enhance the gas permeation and separation performance of the MMMs.

The aim of this study was to investigate the effect of incorporation of amine-functionalized-KIT-6 filler on the CO_2_ and CH_4_ gas permeability and selectivity performance of the MMMs. The KIT-6 was functionalized with (3-Aminopropyl) triethoxysilane (APTES). The functionalized KIT-6 was incorporated into the PSF polymer matrix to fabricate different MMMs. PSF is a type of polymer with good thermomechanical stability, gas permeability, and selectivity [15,16,17,18,19]. The functionalized KIT-6 and the fabricated MMMs were characterized using different analytical techniques. The fabricated MMMs were subjected to CO_2_ and CH_4_ gas permeation and separation at 25 °C.

## 2. Materials and Methods

### 2.1. Chemicals and Materials Used

(3-Aminopropyl) triethoxysilane (APTES, 99.8%) and polysulfone (PSF) were purchased from Sigma Aldrich. Tetrahydrofuran (THF, 99.8%) was purchased from Merck. All chemicals were used without purification.

### 2.2. Functionlization of KIT-6

KIT-6 (1 g) was dispersed in 100 milliliters of dry toluene in a flask. To this solution, 9.0 mmol of APTES was added, and the functionalization of KIT-6 was carried out by refluxing the resulting solution at 80 °C for 24 h [20]. The functionalized KIT-6 sample was filtered, washed with 50:50 distilled water and ethanol mixture, followed by acetone. The obtained sample was dried overnight at room temperature, followed by drying in a vacuum oven at 80 °C for 16 h. The functionalized samples were named as NH_2_KIT-6.

### 2.3. Preparation of the Membranes

Preparation of NH_2_KIT-6/PSF MMMs (which were incorporated with functionalized KIT-6) were conducted following previously reported procedures with some modifications [11,21]. A desired loading (0–4 wt%) of NH_2_KIT-6 was added to 10 mL of THF, followed by ultrasonication for 30 min. The PSF pellet was added and dissolved into the solution for 18 h. Then, the solution was sonicated for 30 min to remove air bubbles, and subsequently was cast on a glass plate using a casting blade with a gap of 200 µm. The glass plate was covered and left for 3 days at room temperature to ensure complete solvent evaporation. The prepared membranes were peeled off and stored in desiccators.

### 2.4. Characterizations of KIT-6, NH_2_KIT-6, and the Membranes

The morphology of the NH_2_KIT-6 filler was analyzed by FESEM (VPFESEM, Zeiss Supra55 VP). NH_2_KIT-6 were subjected to XRD (X’Pert^3^ Powder & Empyrean, PANalytical) scanning for crystalline structure study. Functional groups in KIT-6 and NH_2_KIT-6 were determined by FTIR (Perkin Almer, Frontier). The pore characteristics of KIT-6 and NH_2_KIT-6 were analyzed using N_2_ adsorption–desorption analysis (TriStar II 3020 V1.04) with liquid nitrogen at 77 K. The specific surface area of the sample was calculated by using the Brunauer–Emmett–Teller (BET) method. The mesopore size distribution was determined by using the Barrett–Joyner–Halenda (BJH) method. The morphology of the fabricated membranes was analyzed by FESEM (VPFESEM, Zeiss Supra55 VP). The membranes were also subjected to FTIR analysis (Perkin Almer, Frontier).

### 2.5. Gas Permeation and Separation Studies

The membrane was sealed in a membrane gas cell that was connected to the membrane gas permeation and separation test system. CO_2_ or CH_4_ gas with a purity of 99.99% was fed separately to the membrane gas permeation and separation system. The gas permeation was performed at 25 °C. The pressure difference was regulated to be 5 bar or 7 bar. The permeate flow was measured using a bubble flow meter. The gas permeability, *P*, was calculated using Equation (1):

(1) $$P=\frac{Nl}{\left(P_{f}-P_{p}\right)A}$$

where *l* is the membrane thickness, *N* is the permeate flow, *P_f_* is the feed pressure, *P_p_* is the permeate pressure, and *A* is the membrane area.

The ideal selectivity, *α* of the membrane was calculated as the ratio of CO_2_ gas permeance to CH_4_ gas permeance.

Each gas permeation measurement was repeated three times.

## 3. Results and Discussion

### 3.1. Characterizations of NH_2_KIT-6

**Figure 1:**
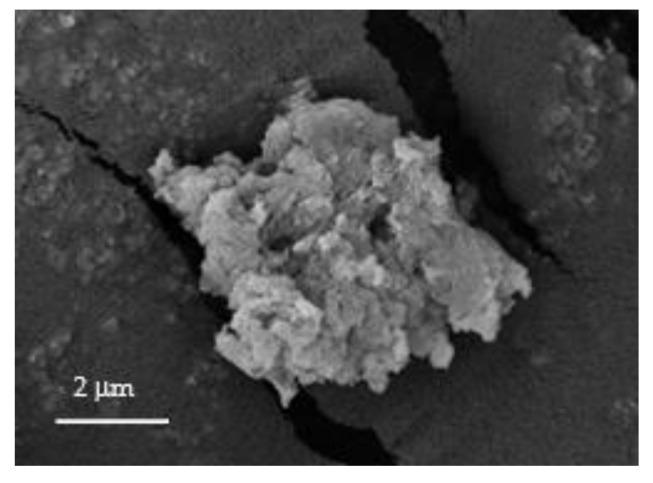
FESEM image of NH_2_KIT-6.

**Figure 2:**
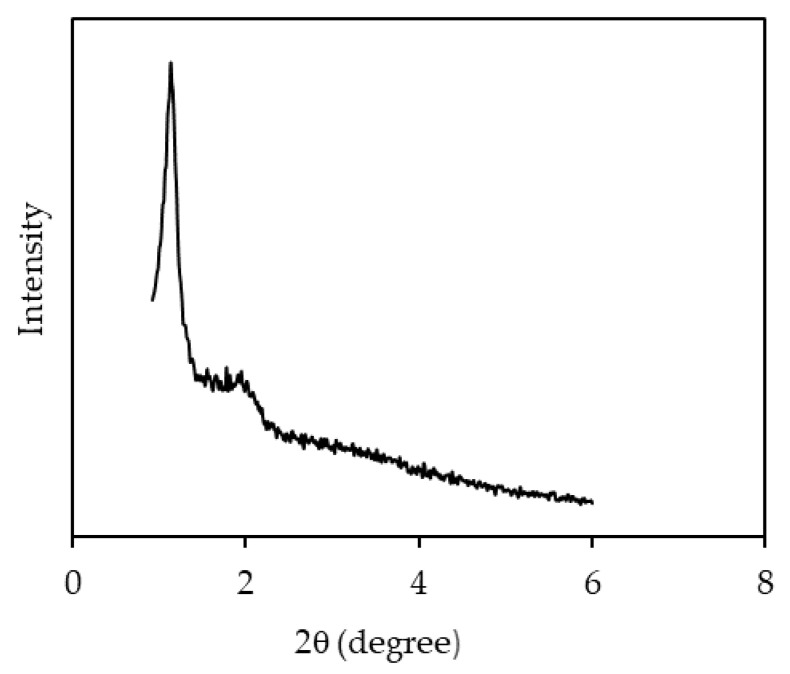
XRD pattern of NH_2_KIT-6.

Figure 1 shows the FESEM image of NH_2_KIT-6. The FESEM image of NH_2_KIT-6 revealed the typical rock-like morphology. Figure 2 shows the XRD pattern of NH_2_KIT-6, which exhibits peak diffractions of about 1.1° at 2θ. This shows that the samples had the ordered mesostructure with a three-dimensional cubic Ia3d symmetry [22]. The XRD pattern of the NH_2_KIT-6 sample in the current project is in agreement with the XRD pattern for KIT-6 reported by Ayad et al. [22].

**Figure 3:**
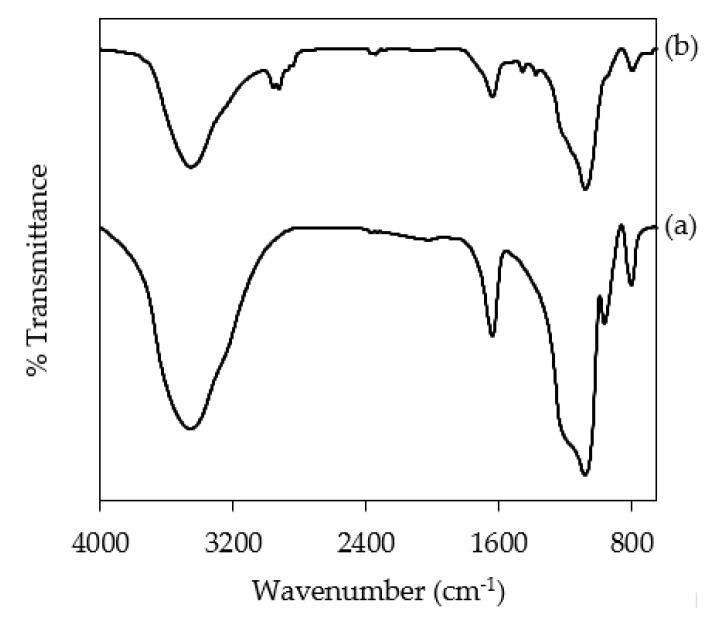
FTIR spectra of (**a**) KIT-6 and (**b**) NH_2_KIT-6.

The FTIR spectra of KIT-6 and NH_2_KIT-6 are shown in Figure 3. The characteristic band at 3464 cm^−1^ indicates the stretching vibration of hydrogen bonding from silanol group y(≡Si−OH) [23]. Furthermore, the characteristic band at 1640 cm^−1^ indicates the O−H bending vibration mode. The anti-symmetric and symmetric stretching vibrations of Si−O−Si groups are observed at characteristic bands at 1083 cm^−1^ and 804 cm^−1^, respectively [24]. It is interesting to observe the characteristic band at 1459 cm^−1^, which is present in the FTIR spectra of NH_2_KIT-6 but is absent in the FTIR spectra of KIT-6. This characteristic band at 1459 cm^−1^ indicates the appearance of N−H bonds in NH_2_KIT-6 and hence prove the successful amine-functionalization of KIT-6 [20,23,25]. In addition, the characteristic band at around 2926 cm^−1^, which indicates the C−H stretching vibration of the organosilane, is observed in the FTIR spectra of NH_2_KIT-6 but is not found in the spectra of KIT-6 [23]. This further proves the effective functionalization of KIT-6.

**Figure 4:**
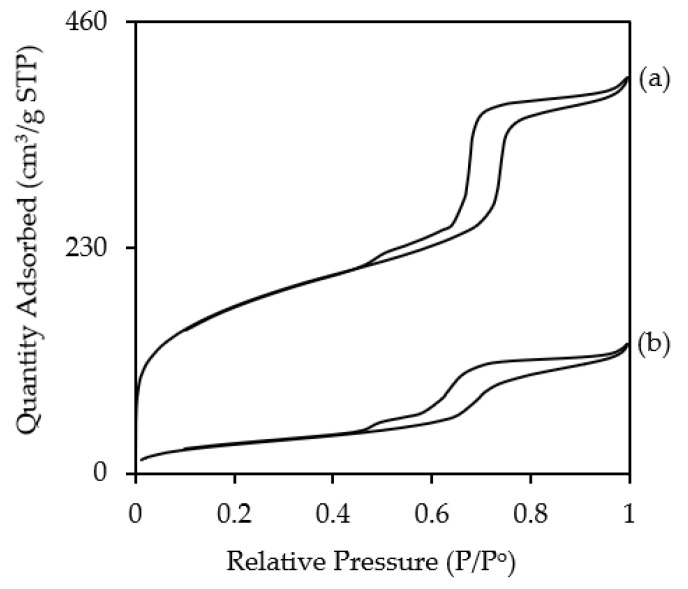
Nitrogen adsorption–desorption isotherm of (**a**) KIT-6 and (**b**) NH_2_KIT-6.

Figure 4 shows the nitrogen adsorption–desorption isotherms of KIT-6 and NH_2_KIT-6. Type IV with a hysteresis loop from the nitrogen adsorption isotherms for KIT-6 and NH_2_KIT-6 is observed, which represents the characteristic of a mesoporous material [4,5,20,26]. According to the IUPAC classification, a type H1 hysteresis loop can be observed from the isotherm; this indicates the characteristic of mesoporous materials and characteristic of material with interconnected large cylindrical pore geometry and a high degree of pore size uniformity [26]. In the current study, NH_2_KIT-6 sample possessed a specific surface area of 108 m^2^/g, a pore volume of 0.18 cm^3^/g, and a pore diameter of 6.50 nm.

### 3.2. Characterizations of the Membranes

**Figure 5:**
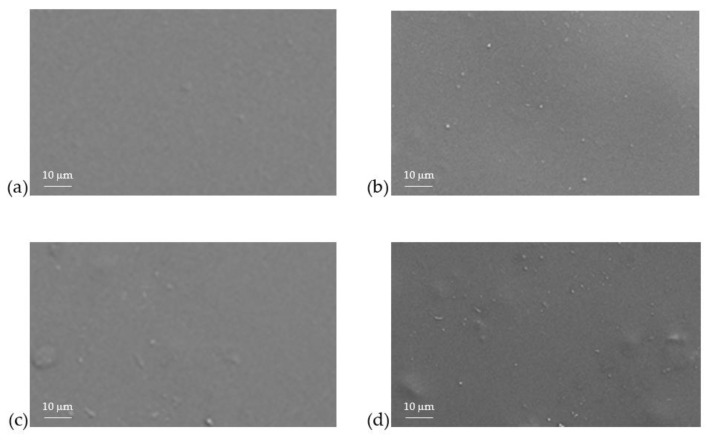
The top view of FESEM images of (**a**) 1% NH_2_KIT-6/PSF, (**b**) 2% NH_2_KIT-6/PSF, (**c**) 3% NH_2_KIT-6/PSF, and (**d**) 4% NH_2_KIT-6/PSF.

**Figure 6:**
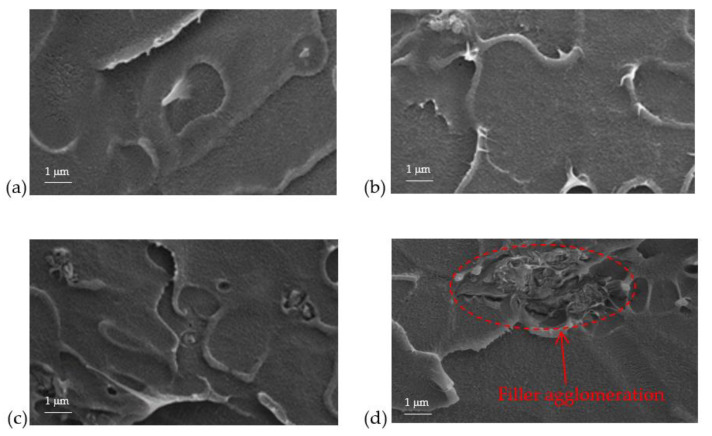
The cross-sectional view of FESEM images of (**a**) 1% NH_2_KIT-6/PSF, (**b**) 2% NH_2_KIT-6/PSF, (**c**) 3% NH_2_KIT-6/PSF, and (**d**) 4% NH_2_KIT-6/PSF.

Figure 5 shows the top view of FESEM images of NH_2_KIT-6/PSF MMMs with different loadings of NH_2_KIT-6. The particles observed indicate that NH_2_KIT-6 was successfully incorporated into the PSF matrix [26]. Figure 6 shows the cross-sectional FESEM images of the NH_2_KIT-6/PSF MMMs. Incorporating up to 2 wt% NH_2_KIT-6 into the MMMs produced MMMs with no void in the matrix. After the functionalization of KIT-6 silica with APTES, the silane backbone of APTES promoted good silica–polymer interactions via van der Waals forces in the MMMs [25,27,28]. However, filler agglomeration was observed when NH2KIT-6 loading in the MMMs was 4 wt%, as highlighted in Figure 6d.

**Figure 7:**
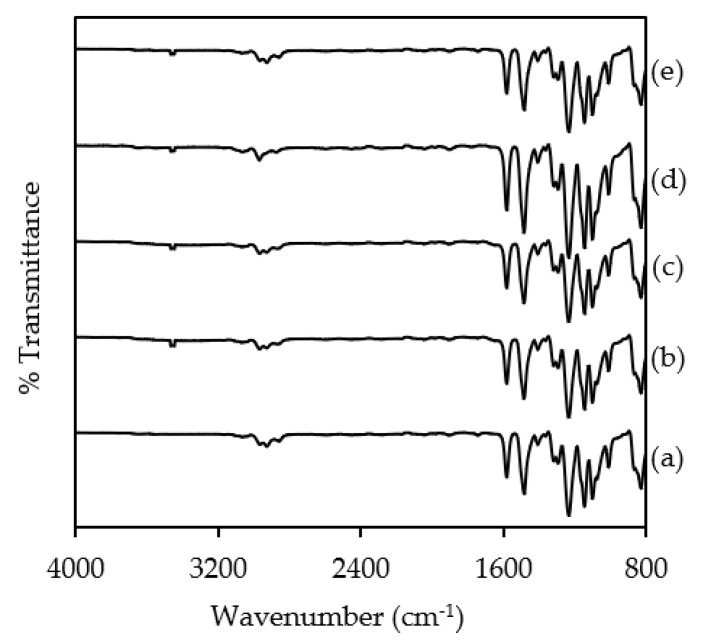
FTIR spectra of (**a**) PSF, (**b**) 1% NH_2_KIT-6/PSF, (**c**) 2% NH_2_KIT-6/PSF, (**d**) 3% NH_2_KIT-6/PSF, and (**e**) 4% NH_2_KIT-6/PSF.

The FTIR spectra of pristine PSF membrane and NH2KIT-6/PSF MMMs are shown in Figure 7. As observed from Figure 7a, the pristine PSF membrane shows C–H rocking at the characteristic band at 831 cm^−1^. The C−C stretching is indicated by characteristic bands at 1012 cm^−1^ and 1103 cm^−1^. The Ar−SO_2_−Ar symmetric stretching is observed at a characteristic band at 1147 cm^−1^, where Ar corresponds to aromatic. On the other hand, the Ar−O−Ar stretching is observed at 1235 cm^−1^ and S=O symmetric stretching is observed at 1294 cm^−1^ [29]. Besides, the characteristic band at 2926 cm^−1^ indicates the C–H stretching vibration [1]. It is interesting to find about the interaction between NH_2_KIT-6 filler and the PSF polymer phase where the characteristic bands of the PSF were retained in the FTIR spectra of the MMMs. This is in agreement with the FTIR analysis reported by Khdary and Abdelsalam [30]. As observed from Figure 7b–e, a small characteristic band at 3464 cm^−1^, which is assigned to the Si–OH group, was observed for the FTIR spectra of four NH_2_KIT-6/PSF MMMs [23]. This indicates the presence of NH_2_KIT-6 in the MMMs. The small characteristic band at 3464 cm^−1^, which is due to the Si–OH group of the NH_2_KIT-6, was not observed in the FTIR spectra of the pristine PSF membrane.

### 3.3. Gas Permeation and Separation Studies

**Figure 8:**
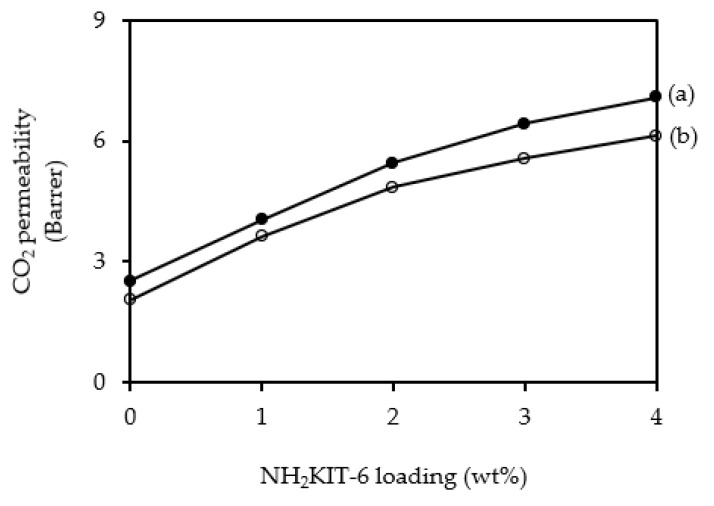
CO_2_ gas permeability of the membranes incorporated with different NH_2_KIT-6 loadings at pressure differences of (**a**) 5 bar and (**b**) 7 bar.

**Figure 9:**
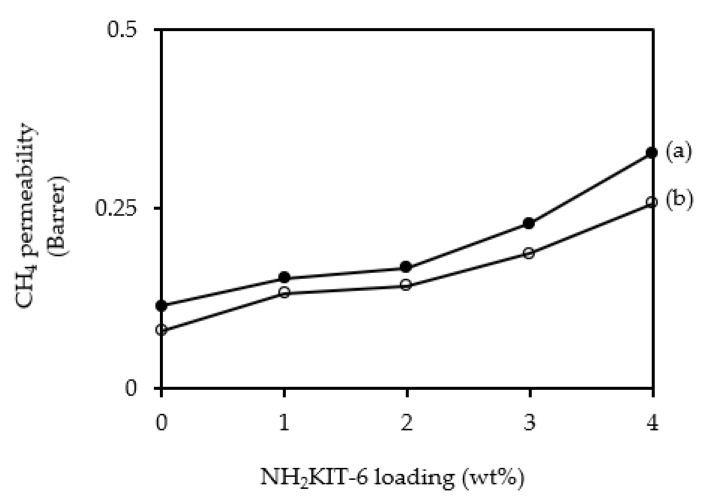
CH_4_ gas permeability of the membranes incorporated with different NH_2_KIT-6 loadings at pressure differences of (**a**) 5 bar and (**b**) 7 bar.

**Figure 10:**
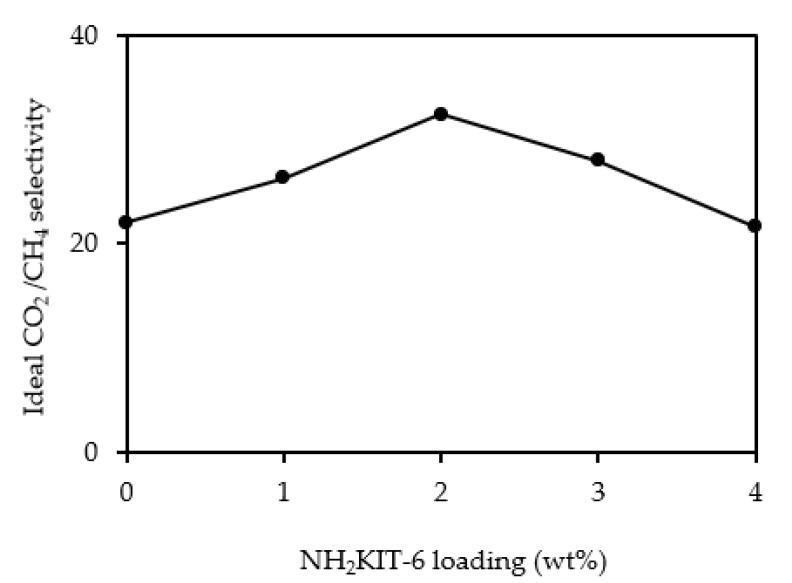
Ideal CO_2_/CH_4_ selectivity of the membranes incorporated with different NH_2_KIT-6 loadings at a pressure difference of 5 bar.

Figure 8 and Figure 9 show the CO_2_ permeability and CH_4_ permeability of the membranes incorporated with different NH_2_KIT-6 loadings. The error for the CO_2_ permeability and CH_4_ permeability was ±5%. As observed in Figure 8, the CO_2_ permeability increased when NH_2_KIT-6 loading in the MMMs was increased. At a pressure difference of 5 bar, an increase of about 47% in ideal CO_2_/CH_4_ selectivity was achieved by incorporating 2 wt% NH_2_KIT-6 into the MMMs. As observed in Figure 8 and Figure 9, the CO_2_ permeability and CH_4_ permeability decreased when the pressure difference increased from 5 to 7 bar. This is a common behavior of glassy polymers [31,32]. Biondo et al. [33] reported a decrease in CO_2_ permeability when the pressure increased for PSF and it was explained that the behavior was typical for a dual-mode model. Figure 10 shows the ideal CO_2_/CH_4_ selectivity of the membranes incorporated with different NH_2_KIT-6 loadings. After the functionalization of KIT-6, the presence of amine groups on NH_2_KIT-6 in the MMMs enhanced the affinity of MMMs toward CO_2_ and hence increased the ideal CO_2_/CH_4_ selectivity of the MMMs [14]. However, further increasing the NH_2_KIT-6 loading from 2 to 4 wt% caused a drop in the ideal CO_2_/CH_4_ selectivity, as observed in Figure 10. This might be due to the filler agglomeration in the MMMs, which started to occur at higher NH_2_KIT-6 loading. In addition, the gas permeability and ideal CO_2_/CH_4_ selectivity of the MMMs incorporated with pristine (unfunctionalized) KIT-6 were reported in our earlier work [34]. The MMMs incorporated with NH_2_KIT-6 in the current study displayed higher ideal CO_2_/CH_4_ selectivity compared to MMMs incorporated with the same loadings of pristine KIT-6 in our earlier work [34]. However, the gas permeation and separation performance of the NH_2_KIT-6/PSF MMMs in the current study fall below the Robeson upper bound [35]. Hence, future research work is still needed in order to further enhance the gas permeation and separation performance of the NH_2_KIT-6/PSF. Table 1 shows the comparison of CO_2_ permeability and ideal CO_2_/CH_4_ selectivity between MMMs from the current study and MMMs incorporated with functionalized silica reported in the literature. The CO_2_ permeability obtained in the current study is quite comparable with the CO_2_ permeability reported in several other studies. As compared with the research works reported in Table 1, a relatively higher ideal CO_2_/CH_4_ selectivity was achieved by incorporating 2 wt% NH_2_KIT-6 filler into the MMMs in the current study.

## 4. Conclusions

In the current study, functionalized KIT-6 (NH_2_KIT-6) was incorporated into a PSF matrix to form MMMs. The presence of particles in the MMMs as observed in the FESEM images indicated the successful incorporation of NH_2_KIT-6 into the MMMs. The CO_2_ permeability and CH_4_ permeability decreased when the pressure difference increased from 5 to 7 bar. MMMs with no void in the matrix were successfully fabricated by incorporating up to 2 wt% NH_2_KIT-6 into the MMMs. Subsequently, an increase of about 47% in the ideal CO_2_/CH_4_ selectivity was achieved by incorporating 2 wt% NH_2_KIT-6 into the MMMs. However, filler agglomeration started to occur in the MMMs when a higher NH_2_KIT-6 loading was incorporated into the MMMs. The ideal CO_2_/CH_4_ selectivity dropped when the NH_2_KIT-6 loading was increased from 2 to 4 wt%.

## Figures and Tables

**Table 1:** Comparison of CO_2_ permeability and ideal CO_2_/CH_4_ selectivity between MMMs from current study and MMMs incorporated with functionalized silica reported in the literature.

Polymer/Filler	Amine Group Used for the Filler Functionalization	Filler Loading (wt%)	CO_2_ Permeability (Barrer)	Ideal CO_2_/CH_4_ Selectivity	Reference
Pebax/MCM-41	Polyethylenimine	5	~87	~23	[14]
PSF/RHS	4-aminophenazone	10	~5.6	~28.1	[1]
PSF/MCM-41	Aminopropyltrimethoxysilane	10	~6.2	~28.0	[13]
PSF/MCM-41	3-aminopropyltriethoxysilane	20	~7.3	~28.1	[11]
PSF/NH_2_KIT-6	(3-Aminopropyl) triethoxysilane	2	~5.4	~32.4	Current study
